# A cluster randomised, 16-week, parallel-group multicentre trial to compare the effectiveness of a digital school-based cognitive behavioural resilience/wellbeing-building intervention targeting emotional and behavioural problems in vulnerable Year 4 primary school children in whole classes, to the usual school curriculum: a study protocol to the “CUES for Schools” trial

**DOI:** 10.1186/s13063-023-07267-3

**Published:** 2023-04-03

**Authors:** S. Jolley, J. Lord, D. Plant, E. Wood, K. Bracegirdle, S. Browning, B. Carter, K. James

**Affiliations:** 1 Department of Psychology, Institute of Psychiatry, Psychology and Neuroscience, King’s College London, De Crespigny Park, London, SE5 8AF UK; 2 Department of Biostatistics and Health Informatics, Institute of Psychiatry, Psychology and Neuroscience, King’s College London, De Crespigny Park, London, SE5 8AF UK; 3 King’s Clinical Trials Unit, Institute of Psychiatry, Psychology and Neuroscience, King’s College London, De Crespigny Park, London, SE5 8AF UK; 4 South London and Maudsley NHS Foundation Trust, Snowfields Adolescent Unit, Mapother House, De Crespigny Park, Denmark Hill, London, SE5 8AZ UK

**Keywords:** Emotional problems, Behavioural problems, CUES for schools

## Abstract

**Background:**

Around 10% of school-aged children experience mental health difficulties. Many more are ‘vulnerable’: experiencing emotional and/or behavioural problems reaching clinical levels, and thus at greatest risk of future mental illness. The trial aim is to evaluate the effectiveness of the CUES for schools programme in reducing emotional and behavioural problems in vulnerable children.

**Methods:**

The “CUES for Schools” study is a multicentre cluster randomised controlled trial in primary schools in south east England. Schools will be randomised to receive the usual school curriculum, or the CUES programme (1:1). We aim to enrol 74 schools (5550 children including 2220 vulnerable children). CUES is a whole-class teacher-facilitated interactive digital cognitive-behavioural intervention, delivered as 24 short (20-min) modules over 12 weeks, targeting emotional/behavioural regulation skills. Children self-report emotional/behavioural problems at baseline, 8, and 16 weeks, and wellbeing and cognitive vulnerability at 0 and 16 weeks. Adverse events are assessed at 8 and 16 weeks. Teachers rate classroom behaviour at baseline and 16 weeks. School senior leadership teams and individual teachers consent to involvement in the study; parents can opt their child out of CUES sessions, assessments, or research. Children can similarly opt out and assent to research participation. The primary objective of this trial is to evaluate the effectiveness of CUES for schools compared to the usual school curriculum in improving emotional/behavioural problems for vulnerable Year 4 (8–9 years old) children at 16 weeks post-randomisation, as measured using a standardised questionnaire designed for primary schools. The secondary objective is to investigate the impact of the CUES for schools programme on both vulnerable and non-vulnerable children on wellbeing and teacher-rated classroom behaviour.

**Discussion:**

The study will show whether CUES for schools is more effective than the usual curriculum in reducing emotional and behavioural problems in vulnerable Year 4 children, and thus reducing the risk of mental health difficulties in later adolescent and adult life. As a digital, teacher-facilitated intervention, CUES for schools can be readily implemented, at minimal cost. If effective, CUES for schools therefore has the potential to reduce the impact of emotional/behavioural difficulties on children’s learning, behaviour, and relationships and the burden of future mental health morbidity.

**Trial registration:**

Trial Registration ISRCTN11445338. Registered on September 12, 2022.

## Administrative information

Note: the numbers in curly brackets in this protocol refer to SPIRIT checklist item numbers. The order of the items has been modified to group similar items (see http://www.equator-network.org/reporting-guidelines/spirit-2013-statement-defining-standard-protocol-items-for-clinical-trials/).

**Table:** 

Title {1}	A cluster randomised, 16-week, parallel-group multicentre trial to compare the effectiveness of a digital school-based cognitive behavioural resilience/wellbeing-building intervention targeting emotional and behavioural problems in vulnerable Year 4 primary school children in whole classes, to the usual school curriculum: a study protocol to the “CUES for Schools” trial
Trial registration {2a and 2b}.	Trial Registration ISRCTN11445338 (September 12th, 2022).
Protocol version {3}	Version 1, 12th September, 2022.
Funding {4}	Funding to conduct the trial is provided by a grant from the Monday Charitable Trust.
Author details {5a}	Jolley S1 + , Lord2,3 + , Plant D4, Wood E1, Bracegirdle4, Browning S4, Carter B2,3&, James K2,3& 1 Department of Psychology, Institute of Psychiatry, Psychology & Neuroscience, King's College London, De Crespigny Park, London SE5 8AF, United Kingdom 2 Department of Biostatistics and Health Informatics, Institute of Psychiatry, Psychology & Neuroscience, King's College London, De Crespigny Park, London SE5 8AF, United Kingdom 3 King’s Clinical Trials Unit, Institute of Psychiatry, Psychology & Neuroscience, King's College London, De Crespigny Park, London SE5 8AF, United Kingdom 4 South London and Maudsley NHS Foundation Trust, Snowfields Adolescent Unit, Mapother House, De Crespigny Park, Denmark Hill, London, SE5 8AZ + These authors contributed equally as joint first author & These authors contributed equally as joint senior author.
Name and contact information for the trial sponsor {5b}	Co-Sponsors South London & Maudsley NHS Foundation Trust South London and Maudsley NHS Foundation Trust (SLaM) R&D Department Room W1.11 Institute of Psychiatry De Crespigny Park London SE5 8AF 020 7848 0251 King’s College, London (KCL) Director of Research Management Director of Administration (Health Schools) Room 1.8 Hodgkin Building Guy's Campus King's College London London SE1 4UL Tel: 020 7848 6960.
Role of sponsor {5c}	The trial sponsor and has delegated responsibility for the overall management of the trial. Queries relating to KCL sponsorship of this trial should be addressed to the KCL Director of Research Management and Innovation or via the trial team.

## Introduction

### Background and rationale {6a}

Three quarters of all adult mental illness onsets before late adolescence (25 years), and half by the age of 15 years, with mental health and substance use accounting for a quarter of disease burden across childhood and adolescence [1, 2]. Globally, around 10% of school-aged children experience mental health problems, such as anxiety and depression; a fifth experience significant emotional and/or behavioural difficulties which impact on learning, behaviour, social relationships, and motivation and increase vulnerability to mental health difficulties in later adolescence and adult life [3]. UK government reports for England suggest higher rates, increasing since 2017 from 10 to 16% [4]. In our recent work, nearly half of 7–10-year olds in London were ‘vulnerable’, herein defined as self-reporting emotional/behavioural problems at levels typical of clinical populations [5, 6].

Reducing vulnerability to future mental ill-health by targeting emotional/behavioural problems in childhood, building resilience and improving wellbeing, has been a global health priority over the last decade [3]. In the context of limited capacity in and access to child and adolescent mental health services (CAMHS), school-based mental wellbeing promotion initiatives have burgeoned, and, in England, become a mandatory part of curricula [7, 8]. Research outcomes are mixed, with the best evidence for cognitive behavioural approaches, delivered to whole classes, which facilitates peer support and normalising without labelling vulnerable children [9–11]. In practice, outcomes continue to be poor, particularly for more vulnerable children: further development of interventions that are readily implementable longer-term is needed [12, 13].

The CUES for schools programme aims to address this need, as a teacher-facilitated, interactive digital platform version of the mental health professional face-to-face delivered CUES-Ed intervention, a cognitive-behavioural whole class approach for 7–10-year olds [5, 6]. CUES is not an acronym in this context; it references the programme content concerning noticing cues to our own emotional state. CUES is designed to reduce emotional/behavioural problems, particularly for vulnerable children, by promoting emotional literacy and emotional/behavioural regulation, both linked with childhood social and academic functioning [14, 15]. CUES also aims to reduce cognitive vulnerability to mental health problems by reducing stigma and promoting flexible thinking and normalising explanations of unusual perceptual experiences, such as seeing or hearing things which others cannot [16].

Development of the CUES programme has required a highly iterative process with children and teachers to ensure a robust and accessible resource. Each learning objective has been translated into a combination of real-life video, animation, plus interactive exercises. As in the original programme, children receive a hard copy workbook and home access to the CUES website.

Service evaluation [5, 6] showed high rates of acceptability by children and teachers and small improvements on whole class wellbeing. Importantly, children identified as vulnerable benefited following CUES-Ed, which motivated its use as our proposed feasibility trial. In this, the CUES for schools programme was found feasible, as we randomised 11 schools (1:1) and 299 schoolchildren to receive CUES, and 6 schools and 419 schoolchildren to the usual curriculum (waitlist control) ([17], ISRCTN12486546]), as well as a suggested signal in vulnerable children consistent with the service evaluation.

The aim of this protocol is to describe the methods underpinning the CUES for schools study. The study aim is to evaluate the effectiveness of the CUES for schools intervention, as an adjunct to the usual school curriculum, compared to the usual school curriculum alone, in reducing emotional and behavioural problems in vulnerable Year 4 school children in England, receiving the intervention as part of a whole class.

### Objectives {7}

#### Primary objective

The primary objective of this trial is to evaluate the effectiveness of CUES for schools compared to the usual school curriculum in improving emotional/behavioural problems for vulnerable Year 4 children at 16 weeks post-randomisation, as measured using the Me & My Feelings total score (M&MF) [17]. This objective relates to the primary sub-population of children meeting the threshold for vulnerability on the emotional and/or behavioural problems subscales of the M&MF (M&MF-E > 9 and/or M&MF-B > 5) at baseline assessment.

#### Secondary objectives

Secondary aims will be to investigate the impact of the CUES for schools intervention on secondary wellbeing outcomes and on teacher-rated classroom behaviour, as well as exploring the effectiveness of CUES for schools across the wider school population (both vulnerable and non-vulnerable).

### Trial design {8}

The design will be a 16-week, multicentre, parallel-group cluster RCT with random allocation of schools to either CUES or the usual curriculum (1:1) to reduce emotional and behavioural problems in vulnerable Year 4 children. The framework of the trial is superiority.

This protocol (Version 1, 12th September 2022) has been written according to the SPIRIT 2013 guidance. The study obtained ethical approval from King’s College London Research Ethics Committee (KCL REC ref. HR/DP-21/22–28,344) on 19th August 2022.

Enrolment will start in October 2022 and is planned to end in February 2023.

## Methods: participants, interventions and outcomes

### Study setting {9}

We will approach the Senior Leadership Teams (SLTs, i.e. the Headteacher and their nominated deputies) of primary schools in inner and outer London and, if needed, extend recruitment to the home counties (the local administrative areas proximal to outer London). Schools will be mainstream (open to all children with no special entry criteria) and funded by local authorities.

### Eligibility criteria {10}

#### Inclusion criteria

The school and child inclusion criteria are shown as follows:

##### School (cluster) inclusion criteria

Funded by the local authority/borough (i.e. a state school, providing education free at the point of delivery to children resident in or with a connection to the local area), providing mainstream education.

##### In London or the home counties

With an intake at Year 4 (children aged 8–9 years) and Year 5 (children aged 9–10 years), so that waitlist control schools, delivering the intervention after completing the 16-week assessment, will definitely have time to deliver the intervention.

##### Child

All children in Year 4 (aged 8–9 years).

#### Exclusion criteria

##### Child

None.

### Who will take informed consent? {26a}

We will approach schools to express interest in participating around 6 weeks before the anticipated randomisation date.

SLTs will be asked for their agreement to participate on behalf of their school. SLTs will consult with teachers of Year 4 children before agreeing to participate, to ensure willingness to deliver the intervention. Once SLTs have consented on behalf of the school, we will liaise directly with teachers to ask formally for their separate consent. Once the SLT and teachers have consented, the school will be considered to be participating in the study.

Information sheets will inform parents of the school’s decision to deliver the intervention as part of a randomised controlled study, and to complete evaluation measures. Parents will be offered the option to remove their child from the CUES teaching and assessments if they wish, by liaising directly with the school.

Parents will be offered the opportunity to opt out of their child’s self-report measures being used for a research purpose. This will be by direct communication with the research team, using an online form. Once parent information sheets have been sent out, children will be told about the study by their teacher, using a video from the study team to standardise the information provided across all schools.

Trained research workers will complete assessments with children and teachers at T0, T1, and T2 (see Table 1).

**Table 1:** Participant timeline

	Engagement with school	Baseline 0-weeks (T0)	Intervention	8-weeks (T1)	16-weeks (T2)
Senior Leadership Team consent	X				
Teacher liaison/consent	X				
Parent letter, opt-out	X				
Child assent		X			
Randomisation			X		
Intervention		➔	➔	➔	➔
Assessment measures					
Primary outcome: Child-rated emotional and behavioural problems (Me and My Feelings)		X		X	X
Secondary outcome: Child-rated wellbeing (Child Outcome Rating Scale)		X			X
Secondary outcome: Teacher-rated outcomes		X			X
Secondary outcome: Child-rated workbook measures		X			X

### Additional consent provisions for collection and use of participant data and biological specimens {26b}

N/a there will be no biological specimens collected in this study.

## Interventions

### Explanation for the choice of comparators {6b}

*The usual school curriculum* for emotional and social learning will be delivered to all children, irrespective of receipt of CUES. The usual school curriculum is nationally set, with limited scope for variation by school. We will ask school senior leadership how they teach these aspects of the curriculum and record what is delivered in the usual curriculum, but will not interfere with usual delivery. In particular, as CUES will not comprise additional hours of teaching, we will record any difference arising in the routine curriculum delivered in intervention and waitlist (WL) control schools.

### Intervention description {11a}

*CUES* comprises seven modules, with 24 lessons delivered over 12 weeks (within a 16-week window), in sessions of 20 min, two or three times/week. Schools will be asked to incorporate CUES into their social and emotional learning provision, so children do not have additional time in the classroom. The programme consists of digital interactive sessions. Teachers guide their class through the sessions using the content and interactivities that are part of the package. The package incorporates branding and characters designed to be appealing and engaging, in a mix of animation and video. Children in the intervention arm will receive CUES straight away. Children in the WL arm will receive CUES later in the term or in the following academic year.

For schools allocated to the CUES arm, teachers will watch the instructional video, and start the CUES programme. CUES delivery should start within 2 weeks of randomisation and proceed at an hour each week. There are 12 h of teaching to deliver within the 16-week window. Measures will be completed at baseline, T1 (8 weeks, primary outcome only) and T2 (16 weeks) in both arms.

The end of the trial will be defined as the last follow-up assessment at T2. WL participants will then receive CUES, at a time suitable in the context of the usual curriculum. This will not be part of the outcomes of the study and children will not be asked to complete any further measures.

### Criteria for discontinuing or modifying allocated interventions {11b}

Not applicable. We will not be reviewing interim data and extensive piloting has revealed no safety issues. Discontinuation would only be at the request of the school, and would be a withdrawal from the study.

### Strategies to improve adherence to interventions {11c}

Teacher adherence to the programme will be assessed by a self-report checklist of completed sessions. The intervention material itself is pre-prepared, so providing it is delivered to the class, adherence has been achieved. Child attendance will be recorded by teachers at T2 as a binary report of whether children attended half or more of the taught sessions, or less than half.

Schools will be randomised to receiving CUES for schools in addition to the usual school curriculum, either now (CUES) or later (WL).

### Relevant concomitant care permitted or prohibited during the trial {11d}

Not applicable. There is no concomitant care, and we are placing no restriction on any school activity in the waitlist condition.

### Provisions for post-trial care {30}

Not applicable. No provision has been made for post-trial care, as we anticipate no need.

### Outcomes {12}

#### Primary outcome

We will use the M&MF [17] total score in vulnerable children at 16 weeks.

This measure comprises 16 items, each rated 0 (best) to 2 (worst), with total scores ranging from 0 to 32. The total score is made up from two subscales — emotional difficulties (M&MF-E, 10 items) and behavioural difficulties (M&MF-B, 6 items) — with children meeting the criterion for being vulnerable based on M&MF-E > 9 and/or M&MF-B > 5. The measure is designed specifically for use in schools to evaluate public health initiatives and has been widely used with children of this age group.

#### Secondary outcomes

##### Vulnerable sub-population

- M&MF-E 10-item subscale (M&MF items 1–10) at 16 weeks. Scores range from 0 to 20, with lower scores indicating more positive outcomes (clinical cut-off > 9).
- M&MF-B 6-item subscale (M&MF items 11–16) at 16 weeks. Scores range from 0 to 12, with lower scores indicating more positive outcomes (clinical cut-off > 5).
- Child Outcome Rating Scale (CORS) [18] 4-item total scale at 16 weeks. This scale is designed to measure wellbeing and distress, with each item rated 0 (worst) to 10 (best) with scores below 32 considered to represent clinical levels of distress/poor wellbeing.
- Child workbook 7-item wellbeing rating scores at 16 weeks. Each item is rated from 0 (worst) to 10 (best), with total scores ranging from 0 to 70.
- Child workbook 8-item cognitive rating scores at 16 weeks. Each item is rated from 0 to 1, with one item rated from 0 to 2. Total scores range from 0 to 9.

##### Whole school population

- M&MF-E 10-item subscale (M&MF items 1–10) at 16 weeks.
- M&MF-B 6-item subscale (M&MF items 11–16) at 16 weeks.
- CORS 4-item total scale at 16 weeks.
- Child workbook 7-item wellbeing rating scores at 16 weeks.
- Child workbook 8-item cognitive rating scores at 16 weeks.
- Teacher ratings of whole class behaviour (ratings I–II) at 16 weeks. This involves estimates of the proportion of the class displaying positive behaviours, rated as the total number of children within the class who ‘often’ or ‘sometimes’ display these behaviours.
- Teacher 4-item coping scale at 16 weeks. Each item is rated from 0 (worst) to 4 (best), with total scores ranging from 0 to 16. This scale is designed to indicate how well the teacher feels they are able to manage emotional upset experienced by children within the classroom.

#### Exploratory outcomes (not formally assessed)

- *Teacher adherence* data will be collected from a self-report checklist.
- *Child attendance* data will be collected from the class teacher.

### Participant timeline {13}

The participant timeline is shown in Table 1.

### Sample size {14}

In our feasibility trial [19], we found an effect size of 0.2. Assuming a type-1 error = 0.05 (two-sided test) with 90% power and an intraclass coefficient (ICC) of 0.025, a total of 68 schools are needed to be included in the analysis population, each with 25 vulnerable children. After accounting for a loss to follow-up at 5% of schools, and then a loss of 15% of vulnerable children, we will randomise 74 schools (1:1) to enrol 2220 vulnerable children.

In typical schools, there are approximately 75 Year 4 children, of which 40% are vulnerable, thus 30 vulnerable children per school. Approximately 5550 children will be enrolled in total, including 2220 vulnerable and 3330 non-vulnerable.

### Recruitment {15}

#### Schools

We will recruit schools through local authority listings, contacting all schools in inner and outer London to inform them of the study and invite an expression of interest before formally liaising with SLTs and teachers for consent. Each school will be approached for SLT consent, with discussion with the research team as needed. Teachers will be approached by their SLT to discuss participation. However, as the SLT is also their management, they will each have a separate discussion with the research team to ensure they have the opportunity to decline participation should they wish to. SLTs will agree, as part of their consent on behalf of the school, to teachers being free to decide to participate or otherwise without this compromising their relationship with their school in any way.

#### Parents

Once SLT and teacher consent are secured, letters will be sent to all parents in the target year group explaining the study and offering the opportunity to opt out of CUES sessions and/or assessments by liaising with the school, or the use of child-reported outcome measures for a research purpose, by liaising with the research team. If parents wish, they can request a discussion with the research team directly using the email provided in the information sheet, or via the school office or teacher.

#### Children

All children will be invited to attend CUES sessions and complete outcomes, unless parents or the children themselves request not to participate. This is because it is important that the parental opt-out process for research use of data does not result in children feeling excluded or stigmatised. Children will give assent for the use of their measures for research. This will be given as privately as possible, again to avoid any stigma. We will only use data when parents have not opted out and children have assented.

## Assignment of interventions: allocation

### Sequence generation {16a}

Randomisation will be carried out following SLT/teacher consent, the sending of parental information sheets, and when teachers and children have completed baseline assessment and prior to the start of the intervention. Cluster randomisation will be managed by an unblinded statistician. We will randomise using covariate-constrained cluster randomisation balancing on school deprivation and school size [20].

### Concealment mechanism {16b}

Randomisation will be managed by the unblinded study statistician. Cluster characteristics needed by the statistician in order to perform the randomisation will be sent through by the trial manager once the school is confirmed as taking part in the study. Allocations will then be sent to the trial manager once all baseline data has been collected from the participants.

### Implementation {16c}

Schools will be randomised once baseline assessments are completed. The member of the study team overseeing the collecting and return of the baseline measures will alert the unblinded study statistician who will send the allocation to the research team. This person will communicate with the school and ensure that appropriate steps are initiated (i.e. teacher induction and onboarding so they can deliver CUES for intervention schools).

## Assignment of interventions: blinding

### Who will be blinded {17a}

Outcome measures are completed by the participants and teachers who cannot be blind to allocation. Researchers facilitating outcome completion will not be blind to allocation, as schools tend to decorate the classroom with CUES materials (e.g. children’s drawings) during delivery. Data processing, however, will be conducted blind to allocation. The senior statistician on the study will remain blind throughout and only ever see pooled data. The trial statistician will remain fully blind until the statistical analysis plan (SAP) is approved by the Trial Steering Committee (TSC) independent statistician, then they will be partially blinded (aware of arms coded A and B).

### Procedure for unblinding if needed {17b}

Not applicable as this is a low-risk study there is no need for an unblinding procedure.

## Data collection and management

### Plans for assessment and collection of outcomes {18a}

Outcome measures (child emotional/behavioural problems, child wellbeing, teacher-rated class behaviour) will be completed by children and teachers at T0, T1 (primary outcome only), and T2, on paper, with support from a research worker online or in-person as required. Measures will be completed as a group in the classroom. Assessments should occur within a 6-week window of their calendar date (up to 2 weeks earlier or up to four weeks later), counted from the day of randomisation. Assessments will be collected by teachers and returned securely by courier to the research team.

### Plans to promote participant retention and complete follow-up {18b}

None.

### Data management {19}

Data will be collected in paper format and will be entered onto and managed using REDCap electronic data capture tools hosted at King’s College, London [21, 22]. REDCap (Research Electronic Data Capture) is a secure, web-based software platform designed to support data capture for research studies, providing (1) an intuitive interface for validated data capture; (2) audit trails for tracking data manipulation and export procedures; (3) automated export procedures for seamless data downloads to common statistical packages; and (4) procedures for data integration and interoperability with external sources.

Teacher adherence, schoolchild attendance, and school reports of usual curriculum delivery will be held in a separate Microsoft Excel [23] database by unblinded members of the research team. Paper forms will be stored securely by the research team until the end of the study (January 2024).

Separate databases will be created for school-level data, class/teacher-level data, child-level data, and allocation. The database will be designed to only accept within range responses. Range and value checks and spot checks against paper copies will be employed to check 20% of entered data.

### Confidentiality {27}

#### Security and backup of data

Data will be stored on password-protected systems in the hosting trust and academic organisations (South London & Maudsley National Health Service Foundation Trust, SLaM and KCL). The allocation database will be accessible only to the lead research worker (who will not conduct post-baseline assessments) and the Chief Investigator (DP) until the study is completed. Outcome data processing will be carried out by researchers who do not have access to allocation, intervention or feedback data. Once all data is entered, cleaned and checked, blind to allocation, the database will be locked. A final database will be returned to the statistician, who will combine with allocation data for analysis. The Chief Investigator will act as custodian for the trial data. The following guidelines will be strictly adhered to:

Child/teacher data will be pseudonymised for the duration of the study and fully anonymised at the end of the study. The fully anonymised data will be kept indefinitely. Fully identifiable personal details will be kept for parents opting out on paper in a locked filing cabinet in a locked or occupied office and on university servers (as responses are returned using a university-based system) until the end of data collection for the study (March 2023).

#### End of the trial

The end of the trial will be defined as a database hard lock. This will be defined as the removal of editing user access for those entering data into the REDCap database.

### Plans for collection, laboratory evaluation and storage of biological specimens for genetic or molecular analysis in this trial/future use {33}

Not applicable. There are no biological specimens collected in this study.

## Statistical methods

### Statistical methods for primary and secondary outcomes {20a}

Prior to the database lock a statistical analysis plan (SAP) will be developed following King’s Clinical Trials Unit (KCTU) Standard Operating procedures (KCTU ST:02 — developing a statistical analysis plan) and approved by the trial team and the independent TSC chair statistician.

Briefly, all statistical analyses will adopt an intention-to-treat principle (ITT) whereby all schoolchildren will be analysed in accordance to the condition in which they were randomised, with this being a modified ITT for the primary outcome to allow for the inclusion of only the vulnerable sub-sample. All analyses will be conducted after data collection has been completed and pre-processed, the SAP has been signed off and the database has been locked. All variables will first be summarised using descriptive statistics (e.g. means and SDs for normally distributed continuous variables, and median and interquartile ranges skewed continuous data), prior to inferential analyses, and M&MF descriptives will be further sub-divided to confirm the proportion of children meeting the criteria for vulnerability at each timepoint.

#### Analysis population

The primary analysis ITT population will include only vulnerable children.

#### Primary outcome analysis

The primary outcome (difference in M&MF scores between CUES and WL at 16 weeks) will be analysed within the vulnerable sub-population using a multilevel, mixed effects linear regression. Here, two random intercepts will be modelled — one at the school level to account for cluster randomisation, and one at the participant level to account for repeated outcome measurement over time. Time, baseline M&MF scores, participant sex, participant age, a dummy variable indicating treatment group, and the balancing variables used for randomisation will be included as fixed effect covariates. A treatment group by time interaction will also be included to allow for the treatment effect to differ at 8- and 16-week follow-ups.

Participants who do not contribute any outcome measurements of the primary outcome at either follow-up time point will not be included in the modified ITT population. Modelling assumptions will be checked and missing outcome data will be handled using maximum likelihood estimation. The framework of the trial is superiority.

#### Secondary outcome analysis

Secondary outcome measures, like the primary outcome, will be analysed using mixed effects linear models in order to account for school-level cluster randomisation. However, as secondary outcomes are measured at one follow-up time period only, no participant-level random effect will be included within the model. Instead, time will be accounted for using fixed effects, with baseline outcome scores, participant sex, participant age, a dummy variable indicating treatment group, the balancing variables used for randomisation, and any additional baseline variables found to predict missingness in the primary outcome variable included as fixed effect covariates. Participants who do not contribute follow-up outcome data will be omitted from these analyses.

### Interim analyses {21b}

There are no interim analyses planned.

### Methods for additional analyses (e.g. subgroup analyses) {20b}

There are no subgroup analyses planned.

### Methods in analysis to handle protocol non-adherence and any statistical methods to handle missing data {20c}

Data will be explored for structural missingness and reported accordingly. The primary population under investigation will be the modified intention to treat (ITT). The ITT population will be defined as all children with at least one post baseline timepoint.

### Plans to give access to the full protocol, participant-level data and statistical code {31c}

Following 1 year after the end of the study researchers may request access to the study data. Any request should be made with a statistical analysis plan addressing a specific research question not answered by the CUES for schools trial. All requests are made to the corresponding author and approved by the CUES trial management group. Statistical code for the primary analysis will be provided upon request.

## Oversight and monitoring

### Composition of the coordinating centre and trial steering committee {5d}

Data monitoring will be the responsibility of the study research lead (SJ), overseen by the trial management group and the steering committee. As data will be collected over a relatively short period of time, there will be no interim analyses. As we do not anticipate risks to participant safety as a direct result of the study and will not be conducting any interim data analysis, we will not convene a separate Data Monitoring Committee (DMC) and will devolve these functions to the trial steering committee (TSC) which will be detailed in the TSC charter. The study will be subject to the standard local and national governance frameworks of SLaM Research & Development, CAMHS clinical services and research coordination, and our ethics committee.

An independent trial steering committee will be established to oversee trial conduct.

### Composition of the data monitoring committee, its role and reporting structure {21a}

We will hold a combined TSC and DMC with a member of the DMC monitoring safety events split by arm at their request and reporting any concerns for the continuation of the study to the TSC during the meetings.

### Adverse event reporting and harms {22}

#### Harms

We will ask teachers to report to the study team any concerns about CUES or the assessment protocol or any other aspect of the study, expressed by teachers themselves, parents, or children. These will be reviewed by the steering committee for severity, and attributability to the study in liaison with the school, and parents if relevant.

#### Withdrawal of participants

Schools will have the right to withdraw from the study at any time up until the start of delivery for any reason. Once delivery has begun, both children and parents will also be involved and a school opt-out will need to take their wellbeing and expectations into account.

### Frequency and plans for auditing trial conduct {23}

The research data will be stored in King’s College London online servers. The Investigator(s) will permit trial-related monitoring, audits, research ethics committee (REC) review, and regulatory inspections by providing the Sponsor(s) and REC direct access to source data and other documents providing this is within the bounds of data protection and the protection of participants’ confidentiality.

### Plans for communicating important protocol amendments to relevant parties (e.g. trial participants, ethical committees) {25}

All substantial major protocol revisions will follow the King’s College Research Ethics Committee revision process and will be approved prior to their implementation. If a revision leads to a substantial change to the study design, e.g. adding an additional time-point, we will repeat our consent process to re-consent parents and children and allow them to opt out to the revised changes.

### Dissemination plans {31a}

Findings will be communicated to participating school SLTs, who will be free to choose the best method for their school for dissemination. We will present the findings of the research at conferences and will publish in peer-reviewed journals. Locally, we will present to services within our Academic Health Sciences Network, where we have close practice and training links.

## Discussion

The CUES for schools study will evaluate an interactive digital platform designed for teacher-facilitated delivery in primary school children. Trial procedures have been shown to be feasible, and in excess of 6000 children have received an expert-delivered version of the programme as part of local service delivery (Plant et al., personal communication).

This study will extend recruitment from inner London to outer London and the home counties. Feasibility study uptake was 11 of 36 eligible schools (30%) with 14 expressing interest. As we are recruiting across nearly 40 boroughs/counties, each with 40–80 eligible schools, we do not anticipate difficulty recruiting to our target of 74 schools and can increase this if insufficient numbers of vulnerable children are enrolled. No teacher declined participation. Our feasibility study employed a parental consent and child assent procedure: 8% of parents and 13% of children declined consent; just over 40% of parents and just over 75% of children returned a form and agreed participation. We anticipate higher parental return rates using an opt-out procedure. Parental and child requests to withdraw from delivery of the programme were low (*n* = 2), similar to in-service delivery, and child and teacher feedback in-service was consistent with acceptability, and safety, with no adverse events reported in the feasibility study, or during in-service delivery [5, 19]. Over 80% of children completing baseline assessments were retained in the feasibility study at follow-up.

Teachers varied in the timeliness of programme delivery in the feasibility study: we have implemented a weekly adherence check for this study to facilitate delivery within the 16-week window.

Data completeness was good (primary outcome missing for 2% of children at baseline, < 1% at follow-up; 0–11% missing data for single items on secondary outcomes, mode = 0), indicating children found the assessments acceptable. Completing all measures at three time points elicited informal feedback of fatigue at repetition. In response, we now ask for only the primary outcome at T1 (8 weeks). Measures are self-report by children and teachers. While standardised measures are designed and well-validated for school use with this age group, there is potential for bias as neither children nor teachers can be blinded to allocation. Thus, the self-report nature of the assessments may offer a limitation. Previous authors have argued that the waiting list control may artificially inflate the between-group difference [24]. It should be noted the same effect was seen in the clinical service evaluation as within the feasibility study point estimate, which may not support this. The same teachers that delivered the CUES for schools lessons to the children carried out the teacher-rated assessments — so this outcome may suffer assessor-rated bias.

The programme is designed to improve emotional/behavioural problems specifically for vulnerable children participating within whole class groups. Service data has shown small, yet consistent improvements, which were replicated in feasibility data. Given the mixed outcome data for vulnerable children taking part in similar programmes in schools to date [9, 10], reliably improving their outcomes is a key public health priority. A significant strength is that the programme can be delivered by teachers without extensive additional training. The training provided is integral to the platform except for a short instructional video on presentation style. Implementability should therefore be high, addressing concerns about the continued rollout of research-based initiatives [13].

While the platform content is set, teachers are free to adapt their own delivery to accommodate local variations in context and cultural needs. The recruitment areas are socio-economically diverse, and the programme is designed to be suitable for all schools irrespective of size or setting.

This is a large randomised controlled trial enrolling all children in consented schools in south east England across a range of socioeconomic areas and should offer generalisability. Given the feasibility work undertaken so far, we anticipate that the study will enrol the majority of children from each school enrolled as well as follow up the majority. There is potential to improve current functioning for children, as well as reducing future mental health burden, should CUES for schools prove effective.

## Trial status

This protocol (Version 1, 12th September 2022) has been written according to the SPIRIT 2013 guidance. The study obtained ethical approval from King’s College London Research Ethics Committee (ref. HR/DP-21/22–28,344) on 19th August 2022.

Enrolment will start in October 2022 and is planned to end in February 2023.

## Data Availability

The final trial dataset will be held by the corresponding author who is the data custodian.

## Abbreviations

ISRCTN: International Standardised Randomised Controlled Trial Number
RCT: Randomised controlled trial
SPIRIT: Standard Protocol ItemsRecommendations for Interventional Trials
M&MF (M&MF-E, M&MF-B): Me & My Feelings, Emotional problems, Behavioural problems subscales, a child-rated outcome measure [17]
SLT: Senior Leadership Team (in schools, usually the headteacher and/or their nominated deputies)
T0, T1, T2: Trial timepoints, baseline (0 weeks), 8 weeks, and 16 weeks, respectively
WL: Waitlist control condition
CORS: Child Outcome Rating Scale, a child-rated outcome measure [18]
ICC: Intraclass coefficient
SAP: Statistical analysis plan
REDCap: Research Electronic Data Capture, data entry platform
SLaM: South London & Maudsley National Health Service Foundation Trust
KCL: King’s College London
KCTU: King’s Clinical Trials Unit
ITT: Intention to treat
DMC: Data Monitoring Committee
TSC: Trial Steering Committee
CAMHS: Child and Adolescent Mental Health Services
REC: Research Ethics Committee
